# Identification and functional analysis of long non-coding RNAs in mouse cleavage stage embryonic development based on single cell transcriptome data

**DOI:** 10.1186/1471-2164-15-845

**Published:** 2014-10-03

**Authors:** Kunshan Zhang, Kefei Huang, Yuping Luo, Siguang Li

**Affiliations:** Stem Cell Translational Research Center, Tongji Hospital, Tongji University School of Medicine, Shanghai, 200092 China; College of Life Sciences, Nanchang University, Nanchang, 330031 China

## Abstract

**Background:**

Long non-coding RNAs (lncRNAs) regulate embryonic development and cell fate decision in various ways, such as modulation of chromatin modification and post-transcription regulation of gene expression. However, the profiles and roles of lncRNAs in early mammalian development have not yet been demonstrated. Here, we reported a comprehensive analysis of mouse cleavage stage embryonic lncRNA profiles based on public single-cell RNA-seq data.

**Results:**

We reconstructed 50,006 high-confidence transcripts in 22,827 loci, and identified 5563 novel lncRNAs from 3492 loci expressed in mouse cleavage stage embryos. These lncRNAs share similar characteristics with previously reported vertebrate lncRNAs, such as relatively short length, low exon number, low expression level and low sequence conservation. Expression profile analysis revealed that the profiles of lncRNA vary considerably at different stages of cleavage stage embryos, suggesting that many lncRNAs in cleavage stage embryos are stage-specifically expressed. Co-expression network analysis suggested many lncRNAs in cleavage stage embryos are associated with cell cycle regulation, transcription, translation and oxidative phosphorylation to regulate the process of cleavage stage embryonic development.

**Conclusions:**

This study provides the first catalog of lncRNAs expressed in mouse cleavage stage embryos and gives a revealing insight into the molecular mechanism responsible for early embryonic development.

**Electronic supplementary material:**

The online version of this article (doi:10.1186/1471-2164-15-845) contains supplementary material, which is available to authorized users.

## Background

The beginning of embryogenesis is cleavage stage. During this stage, a zygote undergoes several rapid rounds of division, and produces a mass of cells within the zona pellucida. Abnormally cleavage, such as multinucleation [1] and asynchrony division [2], will lead to poor developmental competence. In most *in vitro* fertilization (IVF) cases, the evaluation of implantation potential is carried out at cleavage stage embryos, or oocytes, which are based on morphological and/or genetic methods [3, 4]. However, the precision of these methods is far from ideal: only one third of clinical IVF cases successfully result in a pregnancy [5, 6]. Thus, understanding the molecular mechanism underlying cleavage stage development is of paramount importance to the improvement of preimplantation genetic diagnosis (PGD).

Recently, the efforts to characterize cleavage stage embryos have revealed the global gene expression profiles during preimplantation development of mouse embryo [7–14]. A major goal in the study of cleavage stage embryos is to illustrate intricate molecular regulatory networks and to identify key regulators during cleavage stage embryonic development. However, although the expression patterns of messenger RNAs and microRNAs in cleavage stage embryos were discussed in previous studies, the long non-coding RNAs (lncRNAs), which were recently proved to be critical gene regulators of development, are not yet clearly elucidated.

In the past decade, many lncRNAs in variety species were identified via massive parallel sequencing of transcripts (RNA-seq) [15–18]. One limitation of regular RNA-seq is the requirement of large amounts of material and a minimum of 500 pg total RNA input is suggested [19]. Fortunately, several researchers have developed single-cell RNA-seq methods to elucidate gene expression profile in a single cell, such as mouse oocytes and cells from mouse preimplantation embryos [11], mouse bone marrow-derived dendritic cells (BMDCs) [20], human white blood cells [21]. These efforts may help to study new and low abundance lncRNAs expressed in a very limited subset of cell types and reveal the expression variability between individual cells.

Although the functions of most lncRNAs are still unclear, several lncRNAs were found to be involved in cleavage stage embryos development. For example, the well-known X-link lncRNA Xist mediates the X-inactivation since 4-cell stage [22, 23], and Kcnq1ot1, a paternally expressed non-coding RNA expressed since 2-cell stage, regulates the establishment of imprinting in Kcnq1 domain during preimplantation development [24]. These studies suggest that lncRNAs may play an important role in preimplantation development.

Here we report the genome-wide characterization of cleavage stage embryonic lncRNAs, and define a stringent set of 3492 (5,563 transcripts) novel lncRNA genes from single-cell RNA-seq data of mouse cleavage stage embryos. We validated our data set by known genomic features of lncRNAs, including transcript length, exon number, evolutionary conservation and spatiotemporal expression specificity. Weighted gene co-expression network analysis revealed that lncRNAs express in a strong developmental stage-specific manner, and many of them are highly associated with development regulatory genes. Our genome-wide annotation of cleavage stage embryonic lncRNAs may improve our understanding of molecular mechanism that underpin mouse embryogenesis and provide a large number of candidate targets for PGD.

## Results

### Reconstruction of mouse cleavage blastomere transcriptome

**Figure 1:**
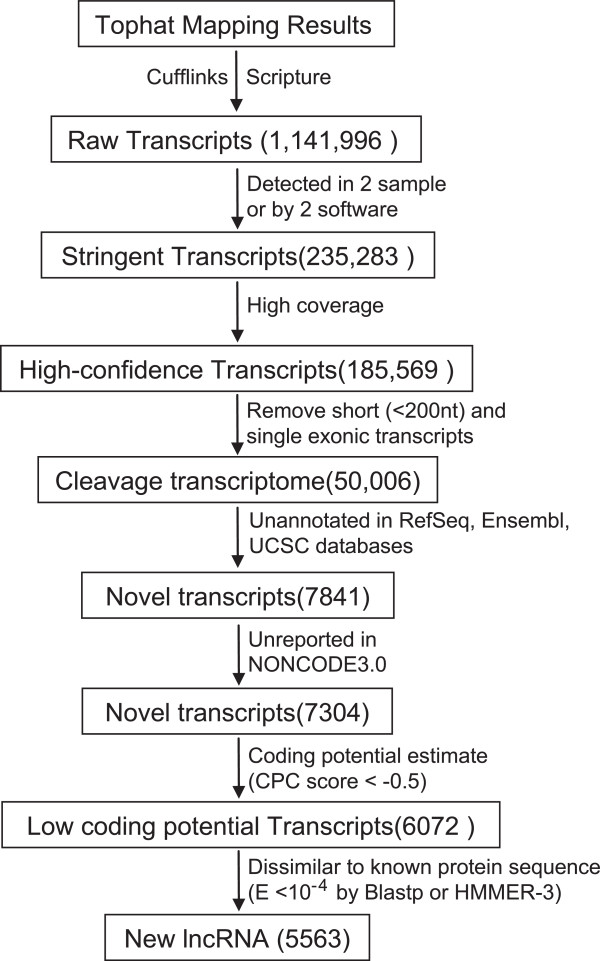
**Overview of cleavage stage lncRNAs identification pipeline.**

To identify lncRNAs involved in mouse cleavage stage embryo development, we first assembled cleavage stage embryonic transcriptome to reexamine the RNA-seq data GSE22182 [11] which include 24 single cell RNA-seq data from four mouse cleavage stages (Figure 1).

Briefly, all reads were aligned to mouse genome (mm9) by using TopHat, a program specifically designed to align RNA-seq reads and discover *de novo* splice junctions [25]. The mappable reads were then assembled into transcripts with two different assemblers, Cufflinks [26] and Scripture [27]. We identified 1,141,996 non-redundant transcripts from 793,423 loci in four embryonic stages.

To eliminate erroneously assembled transcripts, we applied a stringent criteria [28, 29] to identify probable transcripts which should be detected by two assemblers in same sample or identified in at least two individual samples by same assembler and defined a set of 235,283 cleavage stage transcripts. Next, we removed unreliable lowly expressed transcripts by using a learned read coverage threshold similar to previous study [28]. Unlike conventional RNA-seq, the transcripts reconstructed from single-cell RNA-seq are more fragmentary [21], so we applied an integrity threshold of 0.75 to define well-constructed transcripts, which means an annotated transcript will be accepted if 75% of its exon was covered by reads (Method). Since our transcriptomes are reconstructed from a non strand-specific RNA-seq dataset, we determined the direction of transcripts based on the splice junction sequences. All single-exon and short transcripts (<200 nt) were excluded from our dataset. In the end, a set of 50,006 multi-exonic transcripts from 22,827 loci were identified as high-confidence transcripts (Additional file 1). Notably, the vast majority of high-confidence transcripts were detected by both assemblers, suggest that our high-confidence transcriptomes are reliable.

### Identification of 5563 novel lncRNAs in mouse cleavage blastomere

To define novel lncRNAs from our high-confidence transcriptome, we developed a filtering pipeline to remove known mRNAs, potential mRNAs and known ncRNAs (Figure 1). Firstly, we removed all transcripts overlaping exons of known genes recorded in NCBI RefSeq, UCSC and Ensembl databases, resulting in a data set containing 7841 high-confidence transcripts. Then, we compared the genomic coordinates of our predicted lncRNAs with lncRNA database NONCODE (v3.0), and found 537 lncRNAs were collected in NONCODE (v3.0). Thus, the remaining 7304 RNAs are novel RNA transcripts.

Because novel protein-coding transcripts mingle with novel non-coding transcripts in the prediction process, we applied Coding Potential Calculator (CPC) [30] to evaluated protein-coding potential of novel transcripts and remove putative protein-coding transcripts. CPC assess coding potential by considering potential ORFs,the quality and integrity of predicted ORFs, and the homology with known proteins. CPC also parse the output of BLASTX search against known protein sequences by extract features such as number of hits, quality of hits, and concentration of hits in a single ORF. CPC algorithm incorporates these features and returns a CPC score to evaluate coding potential of transcripts. We define an lncRNA with an empirical CPC score threshold (CPC score < -0.5), and 6072 putative noncoding transcripts were retained.

CPC uses only UniRef90 as reference database of protein similarity analysis, and it defines a coding transcript with a relatively stringent parameter (Blastx E-value < 1 × 10^-10^). These might result in loss of some mRNAs with relatively weak similarity to known proteins and therefore cause false positive results in lncRNA discovery. Based on the hypothesis that translation products of mRNAs are likely to have higher similarity to known proteins or protein families than non-coding transcripts, we translated each transcript and estimated their similarity to known proteins or protein families in order to identify mRNAs which were not captured by CPC. By using blastp [31] and HMMER-3 [32], transcripts with an E-value < 10^-4^ that estimated by any of the two algorithms were considered as protein-coding transcripts. Finally, a set of 5563 transcripts from 3492 loci passed all filters and were regarded as novel mouse cleavage stage lncRNAs (Additional files 2 and 3). A quick view of read counts mapping to annotation features such as mRNAs, known lncRNAs and new predicted lncRNAs in this study suggested new lncRNAs highly expressed in zygote genome activation in 2-cell stage (Additional file 4).

### Genomic features of mouse cleavage stage lncRNAs

Previous studies have shown that lncRNAs are shorter, less conserved than protein coding transcripts [26, 28, 29]. Thus we estimated the length, structure, evolutionary conservation of our predicted novel lncRNAs to determine whether mouse cleavage stage lncRNAs are characterized by these features.

**Figure 2:**
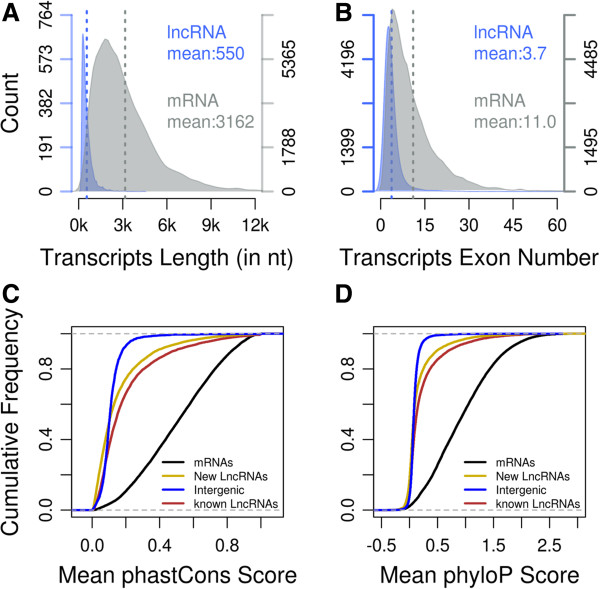
**Genomic features of new predicted lncRNAs. (A)** Length distribution of 27242 coding transcripts and 5563 new predicted lncRNAs. **(B)** Exon number distribution of 27242 coding transcripts and 5563 new predicted lncRNAs. **(C) (D)** Mean phastCons score **(C)** and mean phyloP score **(D)** for 27242 coding transcripts, 35125 known lncRNAs and 5563 new predicted lncRNAs .

We found that the predicted lncRNAs in cleavage stage embryos are fewer in exon number and shorter in length (550 nt and 3.7 exons, on average) than RefSeq protein coding transcripts (3162 nt and 11 exons, on average) (Figure 2A and B). Interestingly, lncRNAs in mouse cleavage stage embryos are shorter in length than lncRNAs in human (~1 kb on average) and zebrafish (1113 nt on average), but more in exon number than lncRNAs in human (2.9 exon on average) [28] and zebrafish (2.8 exons on average) [29].

Although lncRNAs in different species share some conservative genomic features, sequence conservation of lncRNAs is lower than protein-coding genes in different species [18]. Therefore it is difficult to estimate sequence conservation by multiple sequence alignment. Thus, we used two different methods, phyloP [33] and phastCon [34], to estimate the evolutionary conservation of predicted lncRNAs across 30 vertebrate species (Figure 2C and D). We found that our predicted novel cleavage stage lncRNAs are less conserved than protein coding transcripts, but have similar conservation to pre-annotated lncRNAs. Thus, these features of our predicted lncRNAs verified that they are bona fide mouse cleavage stage embryonic lncRNAs which share similar genomic features and evolutionary features with other lncRNAs.

### LncRNA genes and their neighbouring coding genes are globally independent transcripted in mouse cleavage stage embryos

Many lncRNAs are located closely to genes associated with developmental regulatory functions [18, 28, 29]. A subject about lncRNAs is whether lncRNAs transcripted coordinately with neighbouring genes. Therefore we analyzed gene paires formed by lncRNAs and their neighboring genes and identified 4803 lncRNA:coding gene pairs. In these coding genes near new predicted lncRNAs, a significant enrichment (p < 0.05) of morphogenesis and transcription regulation was observed (Additional file 5). Then, we analyzed gene pairs formed by pre-annotated lncRNAs in cleavage stage mouse embryos and their neighbor cording genes and found 3719 RefSeq lncRNA:coding gene pairs. We found that developmental regulatory functions also enriched for their neighbor coding genes (Additional file 6). These results are similar with previous studies in other vertebrates (human [28], cow [35], zebrafish [29]).

Recent studies demonstrated that some lncRNAs can regulate gene transcription in cis [36–39]. We analyzed lncRNAs expressed in cleavage stage mouse embryos, and observed a more correlate expression pattern of lncRNAs and their neighbouring coding genes (mean correlation: 0.268) compare to random coding gene pairs(mean correlation: 0.076) (Additional file 7A; mean p-value = 5.3 × 10^-15^, Kolomogorv-Smirnov Test). On the other hand, coding gene and their coding neighbors (mean correlation: 0.206) also exhibit a relative higher correlated express pattern compare to random coding gene pairs (mean p-value = 2.4 × 10^-6^, Kolomogorv-Smirnov Test). However, lncRNAs:coding gene pairs exhibit a modestly higher correlative expression pattern than coding gene pair (mean correlation:0.268 of lncRNA:coding gene pairs vs mean correlation: 0.206 of coding:coding gene pairs), even there is a significantly different between them (p = 1.97 × 10^-6^, Kolomogorv-Smirnov Test). This observation suggested that the correlation between lncRNAs and their neighbor coding genes are higher than random gene pairs but similar to coding genes pairs.

Previous studies have shown that many lncRNAs are originated within a 4-kb region surrounding the transcription start sites (TSSs) of protein-coding genes and tend to be coordinated with neighbouring protein coding genes [40–42]. We analyzed the distance between TSSs of lncRNAs expressed in mouse cleavage stage embryos (include 5563 new predicted and 4609 annotated lncRNAs) and their neighbouring protein-coding genes. We found that 30.7% (3124/10172) of lncRNAs in cleavage stage embryos were originated within 10 kb from one or more TSSs of protein-coding genes and formed 5148 lncRNA:coding gene pairs. We observed no significant different between lncRNA:coding gene pairs (mean correlation: 0.252) and neighbouring coding pairs (mean correlation: 0.226) (p-value = 0.52, Kolomogorv-Smirnov Test) but both lncRNAs:coding gene pairs and neighbouring coding pairs are more correlated than random coding gene pairs (lncRNA pairs to random pairs, p-value = 1.02 × 10^-9^; neighbouring coding pairs to random pairs, p-value = 3.76 × 10^-8^; both Kolomogorv-Smirnov Test) (Additional file 7B). Further analysis illustrated that the 5' ends of lncRNAs are enriched in a 4 kb region surrounding the TSSs of their neighbouring coding genes (Additional file 7C), which are in agree with previous studies [42].

**Figure 3:**
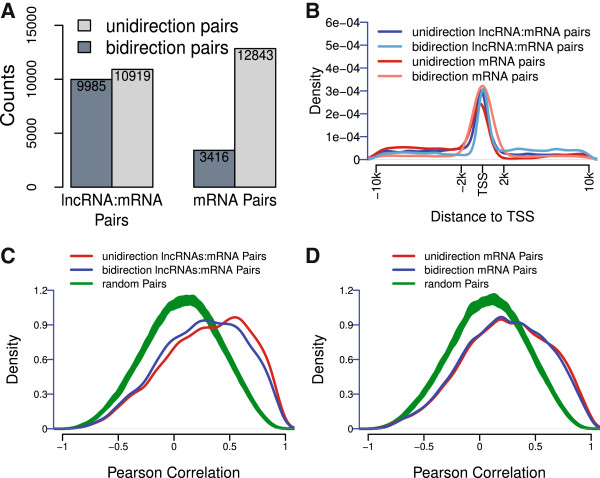
**Bi-directional promoter analysis. (A)** Proportion of different direction in each category of gene pairs. **(B)** Distribution of distance from one TSS to another, in unidirection of lncRNA:coding gene pairs (blue), bidirection lncRNA:coding gene pair (lightblue), unidirection coding gene pairs (red), bidirection neighbor coding gene pairs (lightred). **(C) (D)** Distribution of correlation of TSS adjacent neighbor (distance < 10 kb) in lncRNAs:coding gene pairs **(C)** or neighbouring coding gene pairs **(D)**.

Divergent transcription at promoters of active protein coding genes was considered as an important source of lncRNAs [40–42]. Corresponding to these observations, we observed a higher fraction of bi-directional promoters in lncRNA:coding gene pairs than neighbouring coding gene pairs (Figure 3A). TSSs distance analysis of lncRNA:coding gene pairs revealed that both sense and antisense lncRNAs mainly originate within TSS regions of coding genes (Figure 3B). This distribution is reminiscent of the TSS associated- RNAs (TSSa-RNAs) [40, 41] which peak between -100 nt to -300 nt of antisense TSS. Analysis of all head-to-head genes (include all lncRNAs in this study and all mRNAs in RefSeq database) suggested a common feature of head-to-head genes that the distance of two TSS is range from 0 to -400 nt (Additional file 8), which corrected a previous study [43]. Nevertheless, analysis of neighbor gene expression patterns showed limited differences between lncRNAs:coding gene pairs and neighbouring coding gene pairs in both directions (Figure 3C and D).

Taken together, these analyses revealed that lncRNAs in cleavage stage embryos mainly originated within TSSs region of neighbouring protein coding genes and have coordinated transcription with their neighbors in a similar level of protein coding gene pairs.

### Expression specificity of mouse lncRNAs

**Figure 4:**
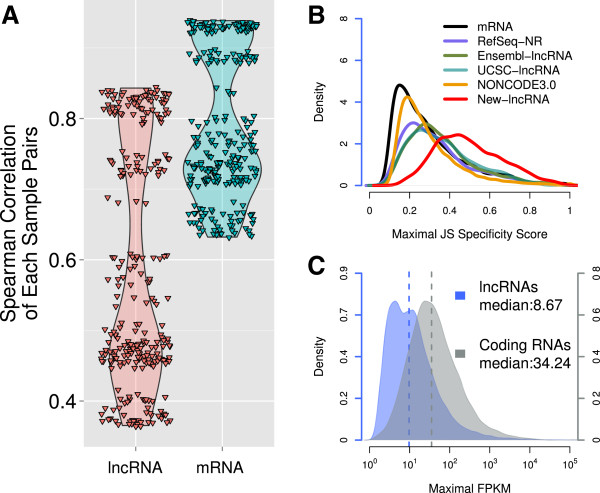
**Temporal specific expression of lncRNAs. (A)** Violinplot indicating distribution of Spearman’s rank correlations between each embryonic sample pairs derived from lncRNAs and mRNAs, respectively. **(B)** Distribution of JSD-based specificity of transcripts in various categories. **(C)** Distribution of maximal expression level of lncRNAs and mRNAs across 24 mouse embryonic RNA-seq data and 33 mouse somatic tissue RNA-seq data.

Previous studies showed that lncRNAs are expressed in a cell-type specific manner and their expression level were significantly lower than that of protein coding transcripts [28, 29]. We calculated the Spearman’s rank correlation coefficients between samples based on lncRNA and protein coding RNA expression data, respectively, and found that the correlation coefficients derived from lncRNA profile data are significantly lower than those derived from mRNA profile data (p-value < 2.2 × 10^-16^, Student T-Test; Figure 4A and Additional file 9) which indicated that lncRNAs are more variable than protein-coding transcripts in early embryonic development. To quantitatively estimate temporal specificity of each transcript during cleavage stage, we applied a Jensen-Shannon distance-based algorithm [28] to calculate temporal expression specificity score of each transcript in 24 single cells from cleavage stage embryos and 33 mouse tissues from ENCODE (GSE39524) (Methods). As expected, our newly identified lncRNAs showed an increased specific expression pattern as compared to protein-coding genes, which is correspond to previous reports [27–29] (Figure 4B). Notably, the specificity of known lncRNAs is modestly higher than protein coding transcripts but lower than lncRNAs identified in this paper. Since most of known lncRNAs were identified from somatic cell lines [27, 44], it is not surprising that known lncRNAs are expressed widely in a variety of somatic organs. In contrast with that, our newly identified lncRNAs are expressed principally in cleavage stage.

We next examine the expression level of lncRNAs and found expression levels of lncRNAs are lower than those of protein coding transcripts (Figure 4C), which agree with the expression patterns of lncRNAs in human and zebrafish [28, 29].

Together, these observations suggested lncRNAs in cleavage stage embryos are expressed in a more temporal-specific manner than protein coding transcripts and lncRNAs identified in somatic tissues. Meanwhile, new identified lncRNAs are expressed in a relatively low level.

### Functions of lncRNAs in mouse cleavage stage embryos

**Figure 5:**
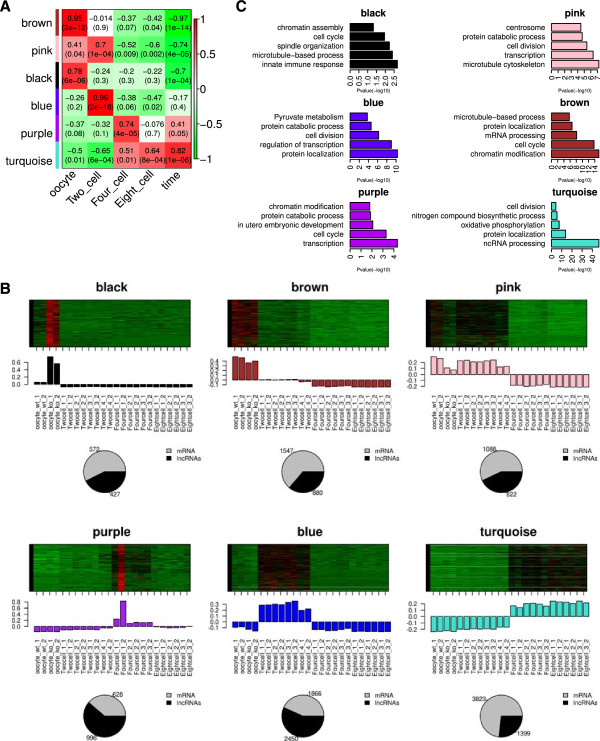
**Function prediction of cleavage stage lncRNAs. (A)** Stage specific co-expression gene modules and their correlation to development stage. Numbers of each square represent correlation of module and development stage, and p-value of each correlation value. Color of each square is correspond to correlation: Positive correlation (Red); Negative correlation (Green); No correlation (White). The column "Time" in the right represents the correlation of each module and entire development process of cleavage stage. Modules with high correlation to time mean overall upregulated during cleavage stage. **(B)** Heatmap in the upper panel is the expression pattern of all genes in this module across all 24 samples. The barplot in the middle panel shows the corresponding module eigengene expression vaule versus each sample. The piechart in the lower panel is the ratio of mRNAs and lncRNAs in module. Number beside the pie chart is the exact number of mRNAs and lncRNAs in this module. **(C)** Function enriched in stage associated modules. Length of bars indicate the significance (-log10 transferred Benjamini-Hochberg adjusted p-value).

To investigate the potential roles of lncRNAs in mouse cleavage stages, we performed weighted gene co-expression network analysis (WGCNA) to associated lncRNAs with mRNAs and predicted their functions based on "guilty-by-association" analysis. By clustering correlated genes together, 24 co-expression gene modules were identified (Additional files 10, 11 and 12). Notably, 6 of 24 modules were highly correlated (correlation > 0.7, p-value <10^-4^) with specific developmental stages or entire process (Figure 5A, Additional file 13).

The functional annotations enriched in each modules indicated that they are clearly functionally related to specific developmental stages (Figure 5B and Additional file 14). In each development stage, especially in 2-cell stage and 4-cell stage, we observed a large number of lncRNAs (Figure 5B), suggesting lncRNA might involved in biological processes which regulate the development of cleavage embryos. Genes in blue module, which is highly relate to 2-cell stage and contains a large fraction of lncRNAs, were enriched in Pyruvate metabolism (KEGG pathway, p-value = 1.8 × 10^-4^), while genes in turquoise module, which is related to development process and contains a relative small fraction of lncRNAs, are enriched in Oxidative phosphorylation (KEGG pathway, p-value = 7.1 × 10^-22^). These results suggest that the lncRNAs in these two modules regulate the switch of predominant nutrient source, which correspond to the previous finding that the predominant nutrient source of cleavage stage embryos is pyruvate in the beginning and switch to glucose around compaction [45].

Notably, black module and pink module are both related to oocyte but differently expressed in wild type and dicer^-/-^ oocytes. The black module, which contains 999 transcripts (572 mRNAs and 427 lncRNAs), is hightly expressed in dicer^-/-^ oocytes, while the pink module, which contains 1910 transcripts (1088 mRNAs and 882 lncRNAs), is highly expressed in WT oocytes and two-cell stage embryos. Dicer is essential for oocyte maturation since the deficient of dicer could lead to misregulation of spindle structure in oocyte [46]. Interestingly, we observed centrosome (p-value = 3.32 × 10^-5^) but not spindle related terms in pink module (normal oocytes). In contrast with this observation, spindle organization (p-value = 5.88 × 10^-3^) was enriched in black module, suggesting the meiotic spindle defects of dicer^-/-^ oocyte may caused by misregulation of post-transcription of spindle related genes. In consideration of the key role of dicer in the metabolism of lncRNAs, we believe that lncRNAs may regulate oocyte maturation and meiosis

Hub genes in modules could reflect the core functions of the modules, so we performed a hub-gene network analysis of each stage-specific modules and found many hub lncRNAs (Additional file 15). The functions of these lncRNAs can be predicted based on their co-expression with hub genes that have known biological functions. For example, in black module, the lncRNAs that co-expressed with Cep78, which is a centrosomal protein, may play roles in regulation of meiosis. In pink module, the lncRNAs that co-expressed with the Rhpn2, which is involved in the organization of the actin cytoskeleton, may play roles in the regulation of spindle organization. Likewise, the lncRNAs in hub gene networks of brown, blue, turquoise, and purple modules may relate to cell cycle, glucose metabolic process, RNA process and protein synthesis. All these networks contain lncRNAs, indicating the key roles of lncRNAs in cleavage stage embryonic development.

## Discussion

The roles of lncRNAs in early embryonic development are intriguing issues with respect to development biology. However, it is difficult to profile mRNA and lncRNA transcriptome in early embryos because of the technical obstacles, such as the analysis of lncRNAs expressed at lower levels and in small amount of cell. Here we, for the first time, illustrated the lncRNA profiles of mouse cleavage stage embryos based on single-cell RNA-seq data. We have identified 5563 novel transcripts from 3492 loci with poor coding potential, which largely expanded the repertoire of lncRNAs. Moreover, we constructed a weighted gene co-expression network and predicted the functions of lncRNAs based on their association with known protein coding genes.

Our newly identified lncRNAs in mouse cleavage-stage embryos shared many characteristics with those in other mammalian species. They are shorter, lower in exon number, lower in expression level and less conserved than protein coding transcripts. Analysis of the co-expression of lncRNAs and their coding neighbors revealed that, globally, the lncRNAs are coordinated to their neighbouring genes in a similar level as the neighbouring coding gene pairs.

Investigation of expression pattern of lncRNAs in cleavage stage embryos elucidated that lncRNAs tend to be expressed in a developmental stage-specific manner. Notably, many lncRNAs are expressed in a very narrow developmental time window and some lncRNAs are restrictedly expressed in an individual cell. These observations suggested that the slight transcriptional changes which may be masked in previously bulk cell analysis can be revealed by single cell transcriptome analysis. Thus, single-cell RNA-seq is a very promising method with high resolution for probe of rare cell types, discovery of low abundant molecules, capture of flash events and detection of weak associations masked in bulk experiments [20, 47].

The highly specific expression patterns of lncRNAs in cleavage stage embryos suggested diverse functions of lncRNAs in early embryonic development. WGCNA based prediction of lncRNA functions associated lncRNAs to different modules with function-known genes, and classified them into different functional groups. Several modules that significantly associated with development stages were investigated and characterized by their functions. For example, lncRNAs in brown module which is highly related to oocyte may function in oocyte meiosis and maturation. In addition, cleavage stage lncRNAs, in modules of brown, blue, purple and turquoise, may regulate cell-cycle based on their module functions. This hypothesis is supported by a recent study which revealed a set of lncRNAs transcribed within cell-cycle promoter of human [48].

The rapid change of lncRNAs during cleavage stage embryonic development also suggests that the stability of lncRNAs is highly regulated. In the procedure of oocyte maturation and oocyte-to-zygote transition, the highly selective degradation of maternal mRNAs is required [49, 50]. An intrinsic question is whether the stability of lncRNAs is regulated by identical or comparable mechanism that regulates stability of mRNAs in cleavage stage development. Study of decapping of lncRNAs in yeast [51] have shown that decapping, as a crucial mechanism in regulating stability of mRNAs and some lncRNAs, is critical for rapid and robust induction of genes which associated with galactose utilization. Thus, the regulatory mechanism of lncRNAs stability may play an essential role in gene expression network in cleavage stage development.

## Conclusions

We provided the first lncRNA profiles of mouse cleavage stage embryos based on single-cell RNA-seq data, and identified 5563 novel lncRNA transcripts from 3492 loci expressed in mouse cleavage stage embryos. These lncRNAs tend to be expressed in a developmental stage-specific manner, many are expressed in a very narrow developmental time window and some are restrictedly expressed in an individual cell. Co-expression network analysis suggested many lncRNAs in cleavage stage embryos are associated with cell cycle regulation, transcription, translation and oxidative phosphorylation to regulate the process of cleavage stage embryonic development. Our results provides the first catalog of lncRNAs expressed in mouse cleavage stage embryos and gives a revealing insight into the molecular mechanism responsible for early embryonic development.

## Methods

### Public data used in this study

Embryonic dataset (GSE22182) was download from Gene Expression Omnibus (GEO) which include four oocyte samples, eight 2-cell stage samples, six 4-cell stage samples, and six 8-cell stage samples. To get somatic dataset, we download 33 somatic tissue data generated by ABI SOLiD platform (GSE39524) from mouse ENCODE project. See Additional file 16 for detail.

### Reads mapping

Reads were aligned to mouse genome (mm9) by the spliced read aligner Tophat (vision 1.4.1) as described in [28]. Briefly, each sample was first aligned to find junctions in each sample (default parameters and ‘max-multihits = 10’, ‘min-anchor-length = 5’). The detected junctions in each sample were pooled as raw junctions for the second round of alignment (default parameters and ‘--no-novel-juncs’). Read counts of new lncRNAs, known lncRNAs and mRNAs in each sample were summarized in Additional file 4, which was performed by HTSeq [52].

### Transcripts assembly

Two different assemblers: Cufflinks (vision 2.0.2) and Scripture (VPaperR3) were hired to assemble transcriptome. The assembly results of these two assemblers were compared by Cuffcompare to identify transcripts detected by both assemblers. Cufflinks-only transcripts were pooled across all samples to identified transcripts occurred in 2 or more samples. Scripture-only transcripts processed ditto. Transcripts < 200 bp or single exonic were excluded.

### Minimal read coverage threshold

To remove bad reconstructed transcripts, alignment artifacts and background expression, transcripts with a maximum coverage below 3.77881 reads per base were eliminated from our transcriptome. To calculate this minimal read coverage threshold, we applied the method described in [28]. The only modification is that we regarded transcripts that recovered 75% of annotation as good reconstructed transcripts. Then we used AUC (area under the curve) to select the optimal threshold of coding and non-coding RNA in Refseq. The final threshold was the average of thresholds for coding and non-coding RNAs.

### Filter of known annotations

We used Cuffcompare to compare our transcripts with those annotations in ① Refseq, ② UCSC gene and ③ Ensemble gene. Transcripts with class code "=" (Complete match of intron chain), "c" (Contained), "j" (at least one splice junction is shared with a reference transcript), "e" (Single exon transfrag overlapping a reference exon), "o" (exonic overlap with a reference transcript), "p" (polymerase run-on fragment), "s" (an intron of the transfrag overlaps a reference intron on the opposite strand) will be removed. The rest of them were considered as novel transcripts. Public annotations used in this study were listed in Additional file 17.

### Analysis of coding potential by CPC

CPC (coding potential calculator) is a SVM-based classifier by comprehensively scoring the characteristics of a transcript including the presence and integrity of predicted ORF, similarity to known protein sequences and conservation of a single frame. We used UniRef90 as known protein reference for CPC analysis. An empirically cutoff (CPC score < -0.5) was used to distinguish mRNA from lncRNA.

### Conservation analysis

The whole genome phyloP score and phastCon score were downloaded from UCSC Genome Browser [53]. Basically, the phyloP/phastCon score of a transcript was defined as the average phyloP/phastCon score of each nucleotide of its exons. Nucleotides which have no phyloP/phastCon score were ignored.

### Neighbouring gene correlation analysis

For genebody neighbouring gene analysis, we defined two genes as neighbours by the minimal distance of genebodies < 10 kb and ignore the direction of two genes. Pearson correlation of two neighbours was calculated with log2-normalization (after addition of 0.05) of raw expression level (FPKM). For TSS distance analysis, the distance of two TSS was calculated. In lncRNA:coding gene pairs, we defined the coordinate (upstream or downstream) of lncRNA TSS by considering the direction of coding gene transcription. In coding:coding gene pairs, the reference TSS was randomly chose.

### Temporal specificity score

The temporal specificity score is defined as 1-(JSdist(p,q)) where p is the density of expression (probability vector of log10(FPKM + 1)) of a given gene across all conditions, and q is the unit vector for that condition (ie. perfect expression in that particular condition), while JSdist is a function that used to calculate pairwise Jensen-Shannon distances between columns in R package "cummeRbund". JS specific score = 1 means a transcript is expressed exclusively in that condition. We use max JS score of a transcript to represent the expression specificity of it.

### Weighted gene co-expression network construction and gene module detection

R package "WGCNA" was used to construct the weighted gene co-expression network [54, 55]. All transcripts passed coverage filter were included in this network. First, a matrix of signed Pearson correlation between all gene pairs was computed. Second, this correlation matrix was raised to power β = 6 to calculate a adjacency matrix. The power of 6 is the soft-threshold of correlation matrix and makes the adjacency network exhibit approximate scale-free topology (R-squared = 0.9). To minimize the noise and spurious associations, the adjacency matrix was transformed to topological overlap matrix (TOM). The matrix 1-TOM was used as the input of average linkage hierarchical cluster. Genes with similar expression pattern were clustered together. We applied the Dynamic Tree Cut algorithm [56] with default parameters to cut the hierarchical tree since modules were defined as branches of the tree. The expression profile of a given module was represented by its first principal component (module eigengene) which can explain the most variation of the module expression levels. Modules with highly correlated module eigengenes (correlation > 0.75) were merged together. The module membership (also known as module eigengene based connectivity, kME) of each genes was calculated by correlating the gene expression profile with module eigengenes, and represents the extent of a gene close to a given module.

### Function enrichment analysis

All function enrichment analyses were performed in DAVID (Database for Annotation, Visualization and Integrated Discovery) [57].

## Electronic supplementary material

Additional file 1: **Transcripts predicted by Cufflinks and Scripture, respectively, in each step of lncRNA identification processes.** (A) Unstrigent transcripts; (B) Strigent transcripts; (C) High-confidence transcripts; (D) Cleavage stage expressed multi-exon and long transcripts. Transcripts predicted only by Cufflinks were shown in green, transcripts predicted only by Scripture were shown in yellow, transcripts predicted by both were shown in purple. (PDF 101 KB)

Additional file 2: **Mouse cleavage embryonic lncRNAs. BED format annotation of mouse cleavage embryonic lncRNAs.** (ZIP 141 KB)

Additional file 3: **Mouse cleavage embryonic lncRNAs.** A Microsoft Excel file contains location and sequence of each transcript. (XLS 4 MB)

Additional file 4: **Read counts in various annotation features of each sample.** The barplot in the upper panel indicate fraction of reads mapped to new lncRNAs (red), known lncRNAs (blue) and coding mRNAs (yellow). The table underneath barplot shows reads mapped in different type of genes. (PDF 47 KB)

Additional file 5: **Neighbor gene functions of new lncRNAs.** Functional terms enriched in 3126 neighbour coding genes which located in 10 kb round of new predicted lncRNAs. (XLS 46 KB)

Additional file 6: **Neighbor gene functions of pre-annotated lncRNAs.** Functional terms enriched in 3028 neighbour genes which located in 10 kb round of pre-annotated lncRNAs. (XLS 712 KB)

Additional file 7: **Neighbouring gene analysis.** (A) Distribution of correlation of neighbouring (genebody distance <10 kb) lncRNA:coding gene pairs (blue), coding gene pairs(red), random gene pairs(100 random permutation; green). (B) Distribution of correlation of neighbor genes TSS (distance between 2 TSS < 10 kb) lncRNA:coding gene pairs(blue), coding gene pairs(red), random gene pairs (100 times random permutation of 20000 coding gene pairs; green). (C) Distribution of distance from one TSS to another, in a lncRNAs:coding gene pair (blue) or in a coding:coding gene pair (red). (PDF 699 KB)

Additional file 8: **Distribution of distance between 2 TSS of neighbour gene pairs.** (A) TSS of lncRNAs which transcribed in identical direction of neighbour coding transcripts; (B) TSS of lncRNAs which transcribed in opposite direction of neighbour coding transcripts; (C) TSS of mRNAs which transcribed in opposite direction of neighbour coding transcripts; (D) TSS of mRNAs which transcribed in opposite direction of neighbour coding transcripts; (PDF 32 KB)

Additional file 9: **Spearman correlation matrix derived from lncRNAs and mRNAs, respectively.** (A) Spearman correlation matrix based on lncRNA expression profile. (B) Spearman correlation matrix based on coding gene expression profile. (PDF 125 KB)

Additional file 10: **WGCNA analysis of expression profile from 24 cleavage stage cells.** Weighted gene co-expression network of 10171 lncRNAs and 10997 mRNAs expressed in cleavage embryos. Dendrogram: hierachical clustering of all transcripts; Upper color panel: module membership of genes; Bottom color panel: scaled gene expression level in 24 cleavage stage cells, (Red) High expression level; (Green) Low expression level; (Black) Median expression level. (PDF 530 KB)

Additional file 11: **Genes and their memberships to each module.** MM is stand for module membership, which is the correlation between a gene and a module. pMM is the p-value of MM. (XLS 20 MB)

Additional file 12: **Module-development stage correlation.** Correlation between development stages and 24 co-expression gene modules defined by WGCNA. (PDF 88 KB)

Additional file 13: **Dynamic change of module Eigengenes of 6 stage specific modules.** Dynamic change of Module Eigengene of 6 stage specific modules across mouse cleavage stage. The color of each line corresponds to module names. (PDF 37 KB)

Additional file 14: **GO and KEGG analysis of stage specific modules.** GO and KEGG terms enriched in 6 development stage specific modules. P-values are Benjamani-Hochberg adjusted. (XLS 575 KB)

Additional file 15: **Hub gene network of stage specific modules.** Hub gene network of stage specific modules, lncRNAs were highlighted in red. Top 100 strength edges and the corresponding nodes (genes) are displayed. (PDF 826 KB)

Additional file 16: **RNA-seq data used in this study.** Sample information of RNA-seq data used in this study. (XLS 16 KB)

Additional file 17: **Public annotations used in this study.** Public annotations used in this study. (DOCX 26 KB)
