# Supplementary material for: Identification and functional analysis of long non-coding RNAs in mouse cleavage stage embryonic development based on single cell transcriptome data
Source: BMC Genomics. 2014 Oct 3;15(1):845. doi: 10.1186/1471-2164-15-845 (PMC4200203; doi:10.1186/1471-2164-15-845)
Supplement: Supplementary file 1 — Additional file 1: Transcripts predicted by Cufflinks and Scripture, respectively, in each step of lncRNA identification processes. (A) Unstrigent transcripts; (B) Strigent transcripts; (C) High-confidence transcripts; (D) Cleavage stage expressed multi-exon and long transcripts. Transcripts predicted only by Cufflinks were shown in green, transcripts predicted only by Scripture were shown in yellow, transcripts predicted by both were shown in purple. (PDF 101 KB) [file 12864_2014_6548_MOESM1_ESM.pdf]

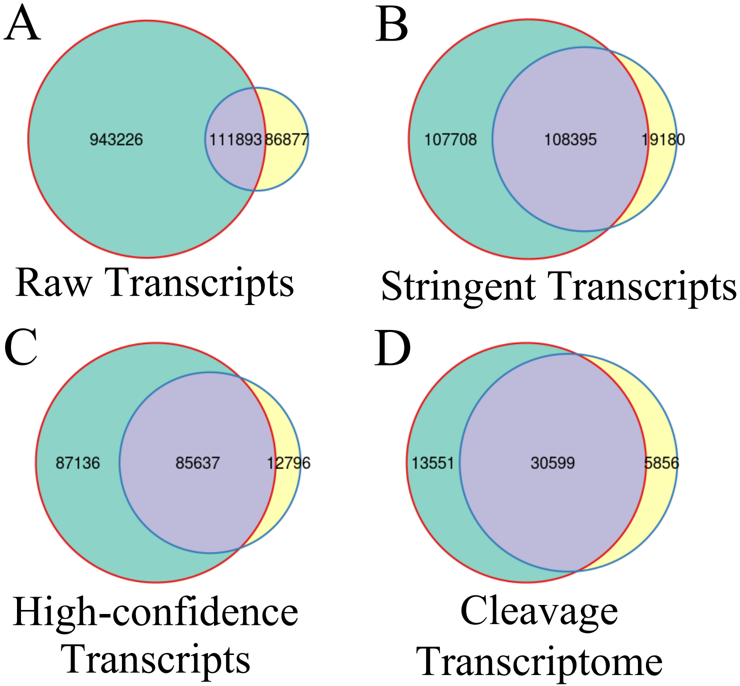
A
943226
111893
86877
Raw Transcripts
B
107708
108395
19180
Stringent Transcripts
C
87136
85637
12796
High-confidence Transcripts
D
13551
30599
5856
Cleavage Transcriptome
