# Supplementary material for: Identification and functional analysis of long non-coding RNAs in mouse cleavage stage embryonic development based on single cell transcriptome data
Source: BMC Genomics. 2014 Oct 3;15(1):845. doi: 10.1186/1471-2164-15-845 (PMC4200203; doi:10.1186/1471-2164-15-845)
Supplement: Supplementary file 4 — Additional file 4: Read counts in various annotation features of each sample. The barplot in the upper panel indicate fraction of reads mapped to new lncRNAs (red), known lncRNAs (blue) and coding mRNAs (yellow). The table underneath barplot shows reads mapped in different type of genes. (PDF 47 KB) [file 12864_2014_6548_MOESM4_ESM.pdf]

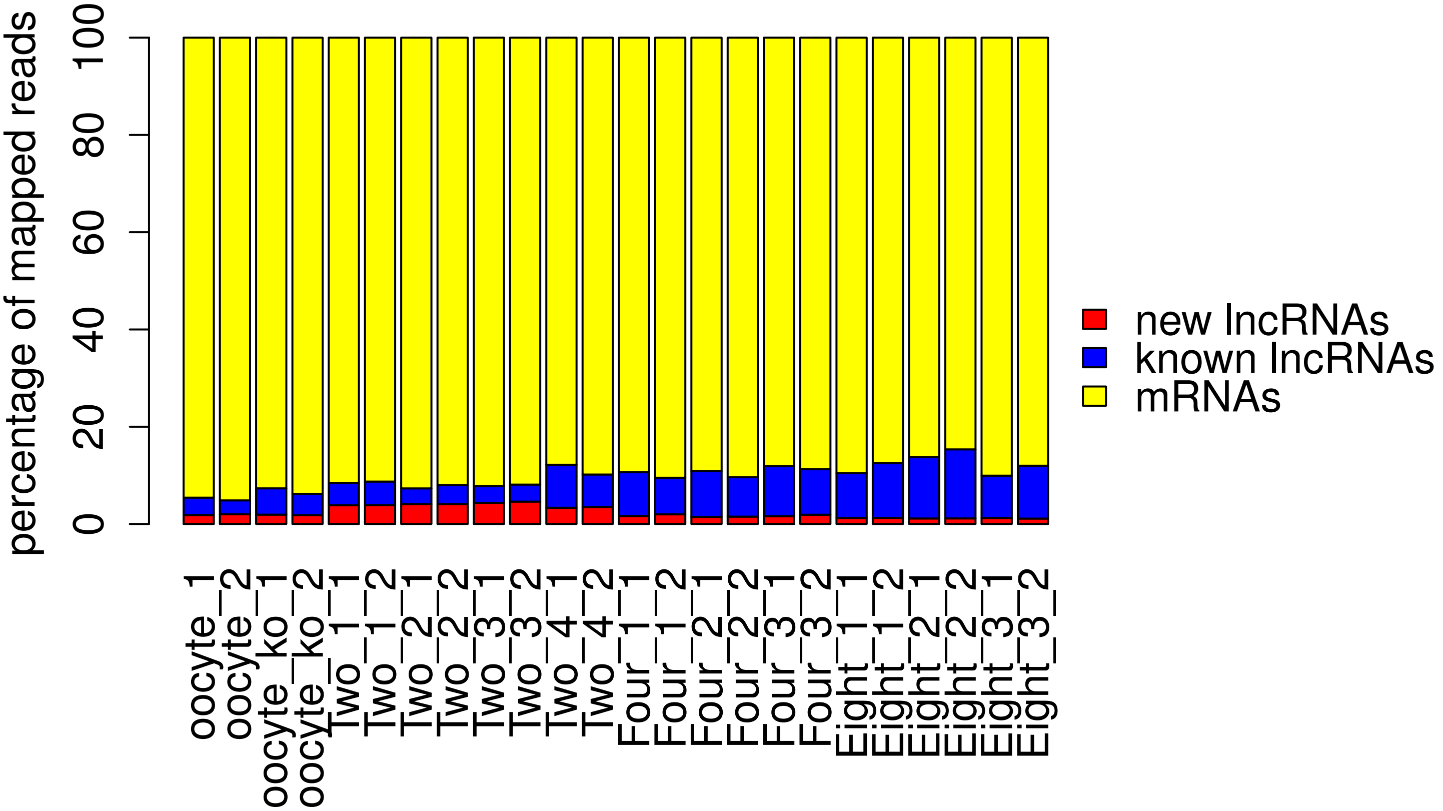

sample	Reads mapped in new lncRNAs	Reads mapped in known lncRNAs	Reads mapped in mRNAs
oocyte_1	198985	391383	10311979
oocyte_2	94902	136925	4549455
oocyte_ko_1	117208	329201	5647694
oocyte_ko_2	105084	258240	5496286
Two_1_1	180518	217174	4305030
Two_1_2	183169	229661	4320867
Two_2_1	169509	134365	3851701
Two_2_2	202570	196638	4578518
Two_3_1	131521	104688	2785874
Two_3_2	266908	203313	5336902
Two_4_1	124564	329869	3276144
Two_4_2	155083	296949	3994165
Four_1_1	68801	378675	3749666
Four_1_2	148438	564023	6779121
Four_2_1	31485	204776	1929961
Four_2_2	101435	542651	6063727
Four_3_1	38231	250774	2138460
Four_3_2	146525	722254	6833691
Eight_1_1	84902	630842	6130614
Eight_1_2	59456	530574	4116207
Eight_2_1	85888	955551	6522671
Eight_2_2	74826	927770	5533609
Eight_3_1	75753	535057	5537155
Eight_3_2	63628	620754	5027652
