# Supplementary figures and images for: Identification and functional analysis of long non-coding RNAs in mouse cleavage stage embryonic development based on single cell transcriptome data
Source: BMC Genomics. 2014 Oct 3;15(1):845. doi: 10.1186/1471-2164-15-845 (PMC4200203; doi:10.1186/1471-2164-15-845)

**A**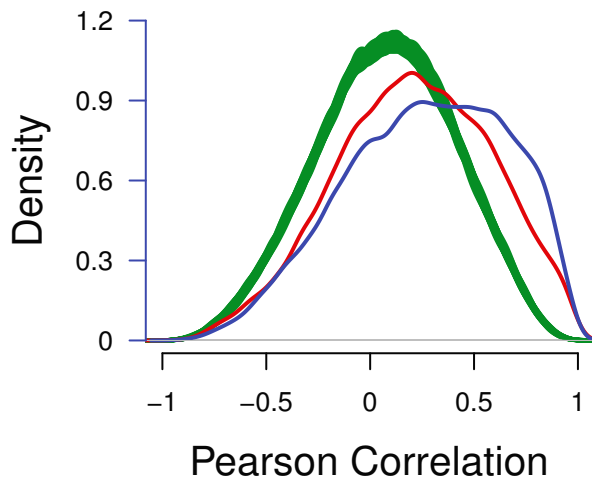**B**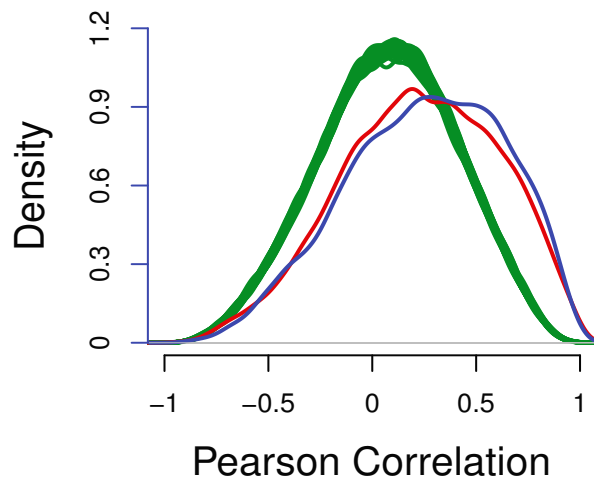**C**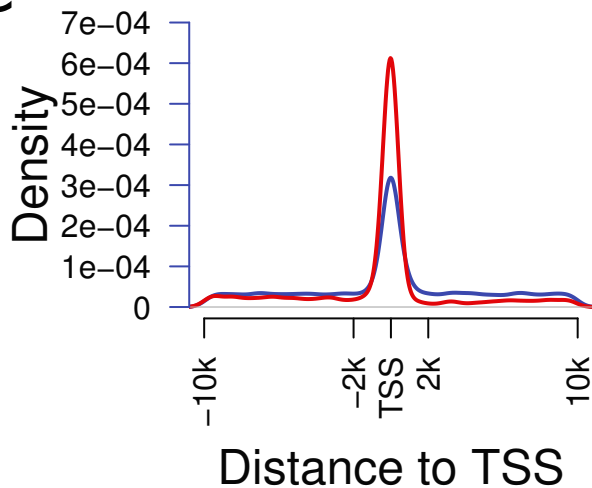

IncRNAs:mRNA Pairs  
mRNA Pairs  
Random mRNA Pairs

Supplement: Supplementary file 7 — Additional file 7: Neighbouring gene analysis. (A) Distribution of correlation of neighbouring (genebody distance <10 kb) lncRNA:coding gene pairs (blue), coding gene pairs(red), random gene pairs(100 random permutation; green). (B) Distribution of correlation of neighbor genes TSS (distance between 2 TSS < 10 kb) lncRNA:coding gene pairs(blue), coding gene pairs(red), random gene pairs (100 times random permutation of 20000 coding gene pairs; green). (C) Distribution of distance from one TSS to another, in a lncRNAs:coding gene pair (blue) or in a coding:coding gene pair (red). (PDF 699 KB) [file 12864_2014_6548_MOESM7_ESM.pdf]

**A****Sense lncRNAs**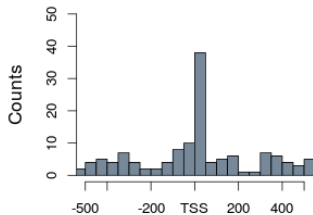**B****Antisense lncRNAs**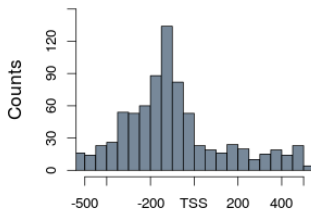**C****Sense Coding Genes**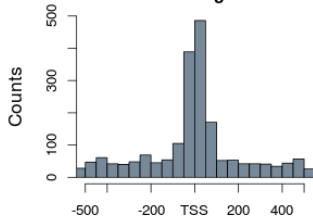**D****Antisense Coding Genes**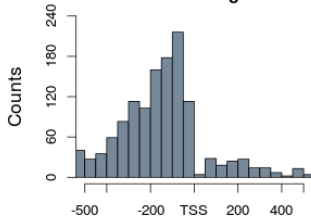

Supplement: Supplementary file 8 — Additional file 8: Distribution of distance between 2 TSS of neighbour gene pairs. (A) TSS of lncRNAs which transcribed in identical direction of neighbour coding transcripts; (B) TSS of lncRNAs which transcribed in opposite direction of neighbour coding transcripts; (C) TSS of mRNAs which transcribed in opposite direction of neighbour coding transcripts; (D) TSS of mRNAs which transcribed in opposite direction of neighbour coding transcripts; (PDF 32 KB) [file 12864_2014_6548_MOESM8_ESM.pdf]

A

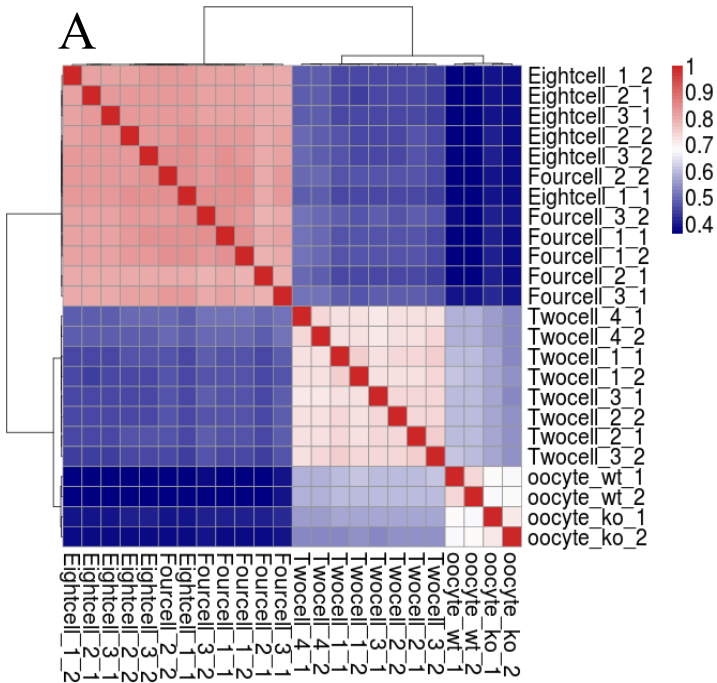

B

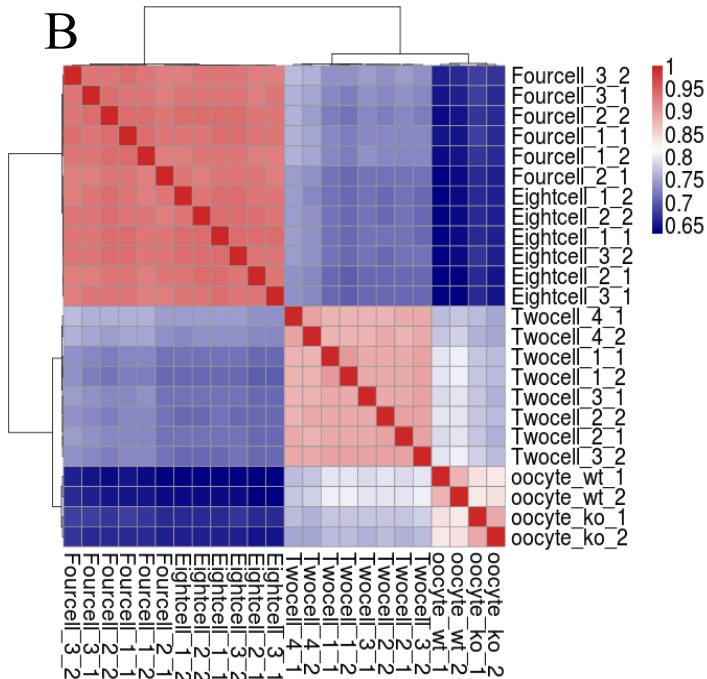

Supplement: Supplementary file 9 — Additional file 9: Spearman correlation matrix derived from lncRNAs and mRNAs, respectively. (A) Spearman correlation matrix based on lncRNA expression profile. (B) Spearman correlation matrix based on coding gene expression profile. (PDF 125 KB) [file 12864_2014_6548_MOESM9_ESM.pdf]

# Cluster Dendrogram

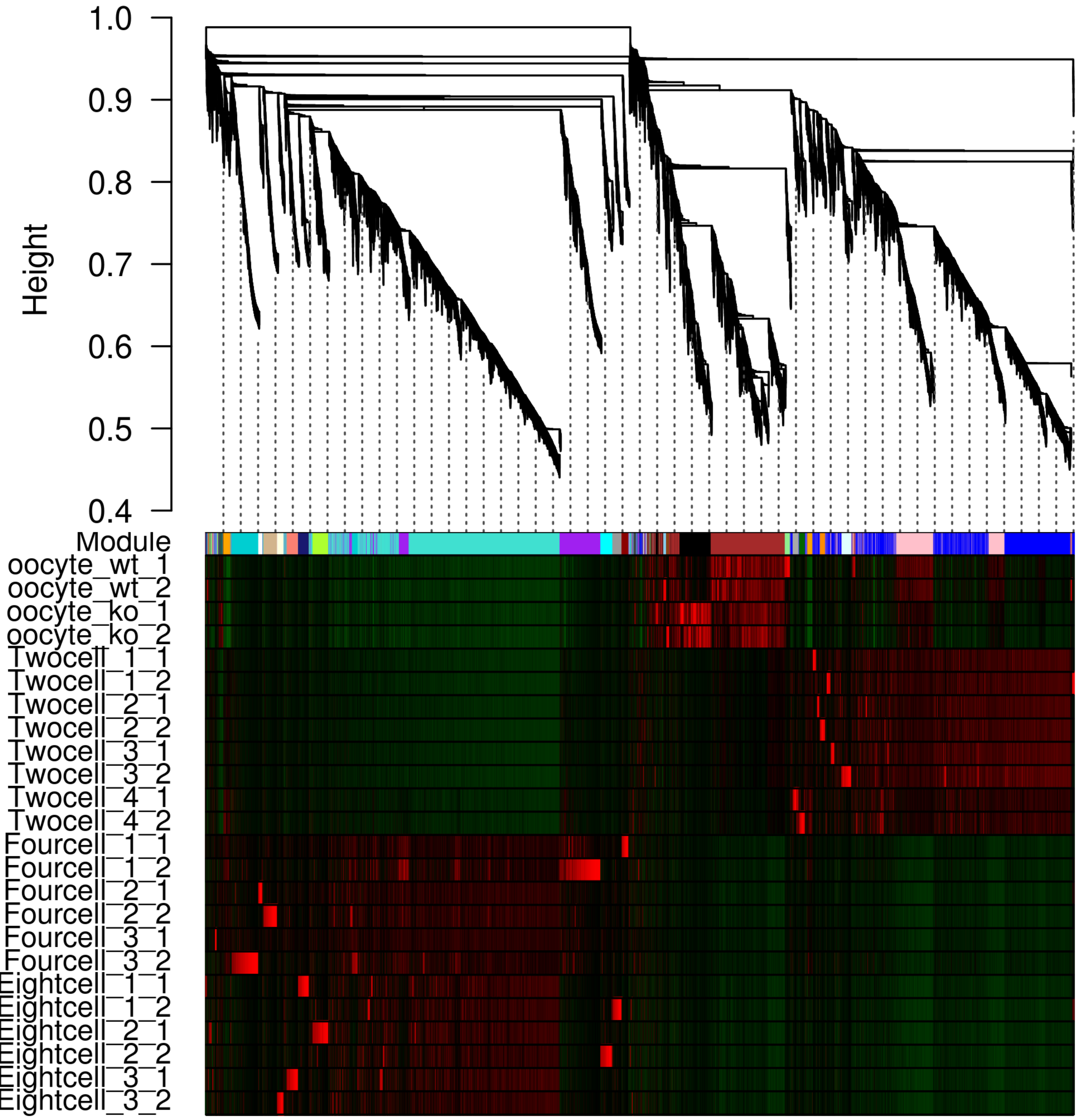

Supplement: Supplementary file 10 — Additional file 10: WGCNA analysis of expression profile from 24 cleavage stage cells. Weighted gene co-expression network of 10171 lncRNAs and 10997 mRNAs expressed in cleavage embryos. Dendrogram: hierachical clustering of all transcripts; Upper color panel: module membership of genes; Bottom color panel: scaled gene expression level in 24 cleavage stage cells, (Red) High expression level; (Green) Low expression level; (Black) Median expression level. (PDF 530 KB) [file 12864_2014_6548_MOESM10_ESM.pdf]

# Module-trait relationships

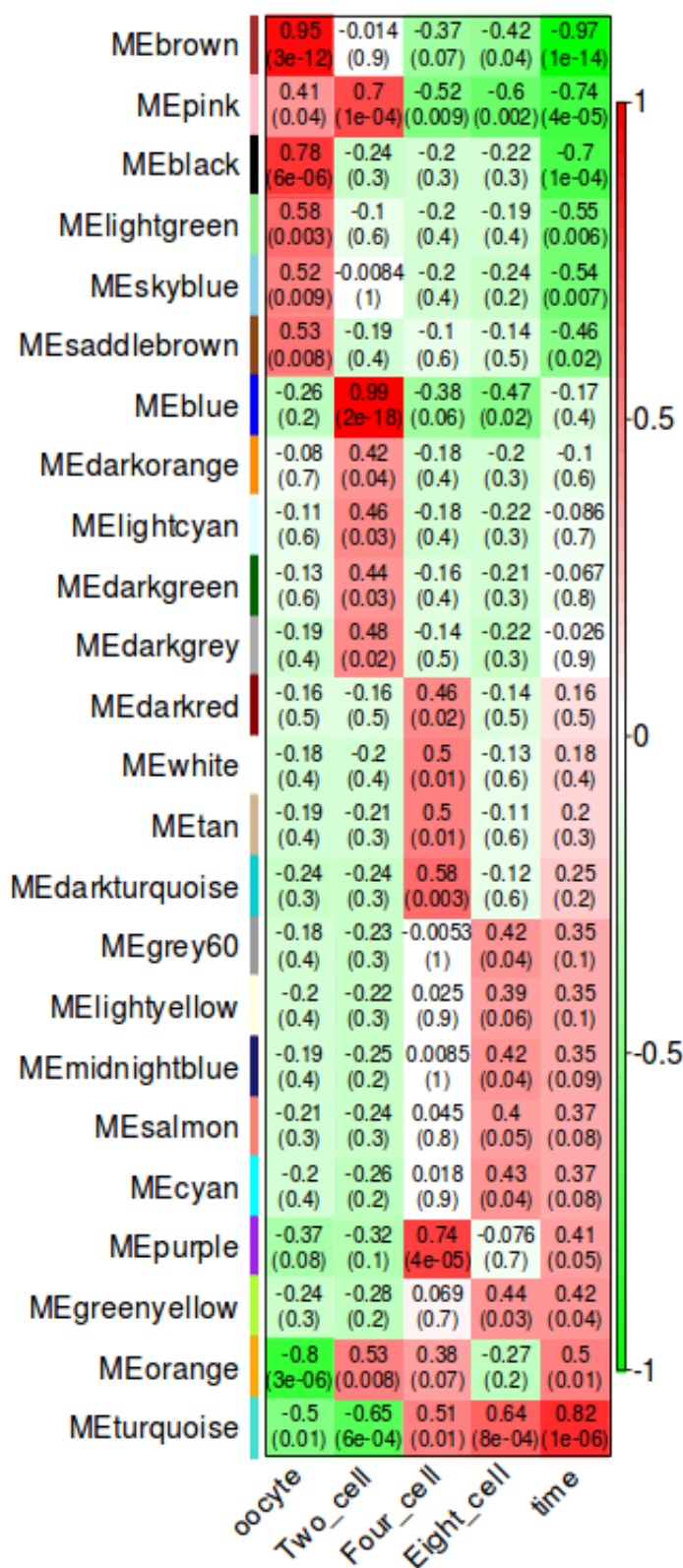

Supplement: Supplementary file 12 — Additional file 12: Module-development stage correlation. Correlation between development stages and 24 co-expression gene modules defined by WGCNA. (PDF 88 KB) [file 12864_2014_6548_MOESM12_ESM.pdf]

Module Eigengene

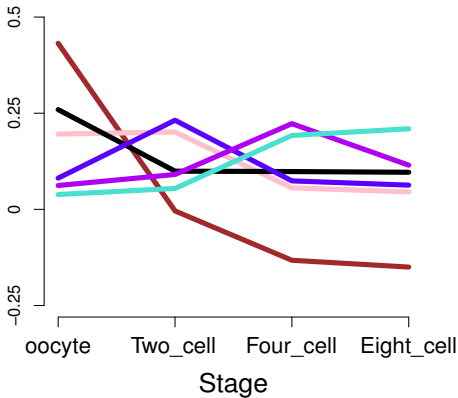

Supplement: Supplementary file 13 — Additional file 13: Dynamic change of module Eigengenes of 6 stage specific modules. Dynamic change of Module Eigengene of 6 stage specific modules across mouse cleavage stage. The color of each line corresponds to module names. (PDF 37 KB) [file 12864_2014_6548_MOESM13_ESM.pdf]
