# Supplementary material for: Identification and functional analysis of long non-coding RNAs in mouse cleavage stage embryonic development based on single cell transcriptome data
Source: BMC Genomics. 2014 Oct 3;15(1):845. doi: 10.1186/1471-2164-15-845 (PMC4200203; doi:10.1186/1471-2164-15-845)
Supplement: Supplementary file 15 — Additional file 15: Hub gene network of stage specific modules. Hub gene network of stage specific modules, lncRNAs were highlighted in red. Top 100 strength edges and the corresponding nodes (genes) are displayed. (PDF 826 KB) [file 12864_2014_6548_MOESM15_ESM.pdf]

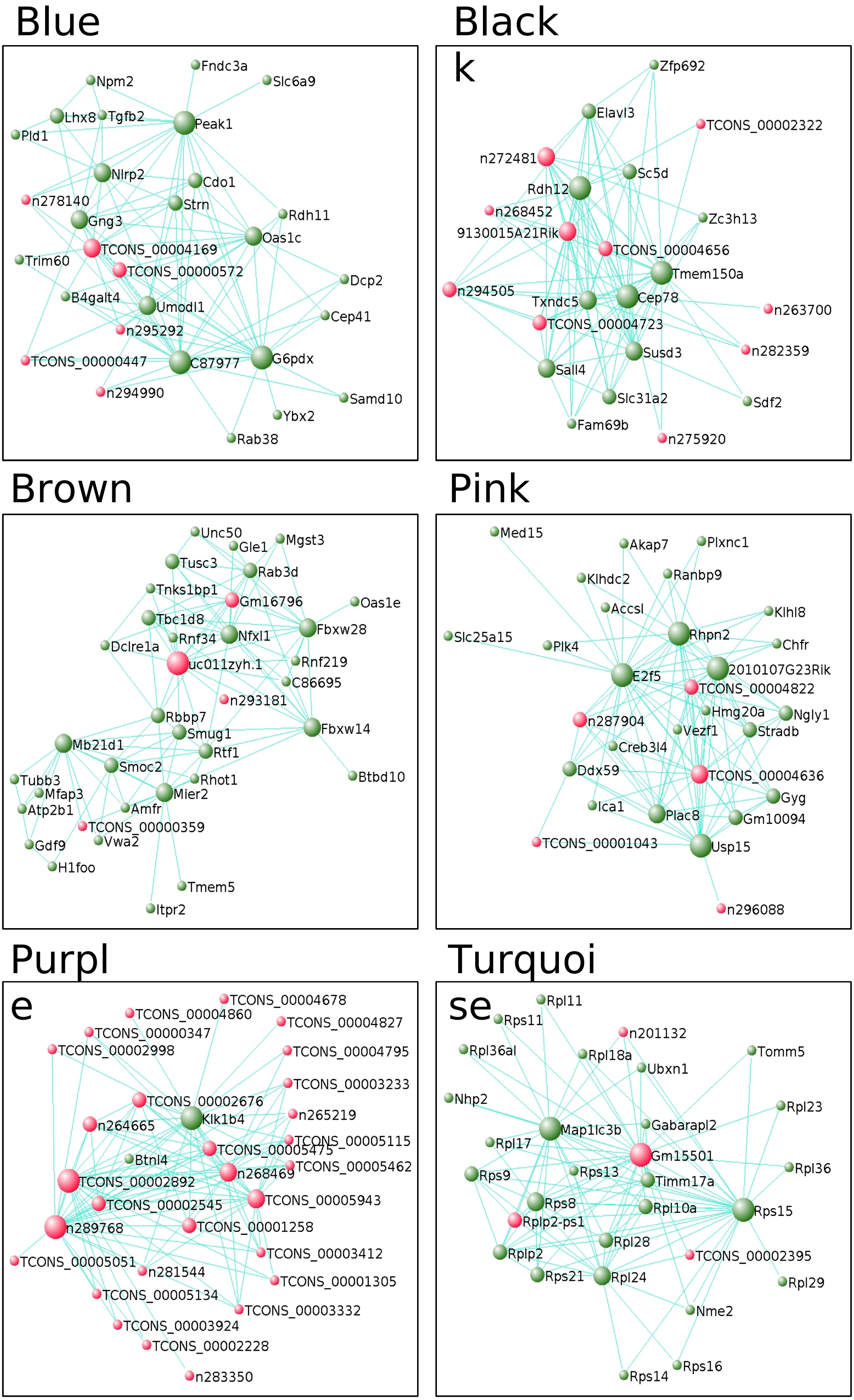
Blue
Fndc3a
Npm2
Slc6a9
Lhx8
Tgfb2
Peak1
Pld1
Nlrp2
Cdo1
n278140
Strn
Rdh11
Gng3
Oas1c
TCONS_00004169
Trim60
TCONS_00000572
Dcp2
B4galt4
Umodl1
Cep41
n295292
TCONS_00000447
C87977
G6pdx
n294990
Samd10
Ybx2
Rab38
Black
k
Zfp692
Elavl3
TCONS_00002322
n272481
Sc5d
Rdh12
n268452
Zc3h13
9130015A21Rik
TCONS_00004656
Tmem150a
n294505
Txndc5
Cep78
n263700
TCONS_00004723
n282359
Susd3
Sall4
Slc31a2
Sdf2
Fam69b
n275920
Brown
Unc50
Mgst3
Gle1
Tusc3
Rab3d
Tnks1bp1
Gm16796
Oas1e
Tbc1d8
Fbxw28
Rnf34
Nfxl1
Dclre1a
uc011zyh.1
Rnf219
C86695
n293181
Rbbp7
Fbxw14
Smug1
Mb21d1
Rtf1
Smoc2
Tubb3
Rhot1
Btbd10
Mfap3
Mier2
Atp2b1
Amfr
TCONS_00000359
Vwa2
Gdf9
H1foo
Tmem5
Itpr2
Pink
Med15
Akap7
Plxnc1
Klhdc2
Ranbp9
Accsl
Klhl8
Slc25a15
Plk4
Rhpn2
Chfr
E2f5
2010107G23Rik
TCONS_00004822
Hmg20a
Ngly1
n287904
Vezf1
Stradb
Creb3l4
Ddx59
TCONS_00004636
Gyg
Ica1
Plac8
Gm10094
TCONS_00001043
Usp15
n296088
Purpl
e
TCONS_00004678
TCONS_00004860
TCONS_00004827
TCONS_00000347
TCONS_00002998
TCONS_00004795
TCONS_00003233
TCONS_00002676
Klk1b4
n265219
n264665
TCONS_00005115
TCONS_00005475
Btnl4
TCONS_00005462
TCONS_00002892
n268469
TCONS_00002545
TCONS_00005943
n289768
TCONS_00001258
TCONS_00003412
TCONS_00005051
n281544
TCONS_00001305
TCONS_00005134
TCONS_00003332
TCONS_00003924
TCONS_00002228
n283350
Turquoi
se
Rpl11
Rps11
n201132
Rpl36al
Rpl18a
Tomm5
Ubxn1
Nhp2
Rpl23
Gabarapl2
Map1lc3b
Rpl17
Gm15501
Rps9
Rps13
Rpl36
Timm17a
Rps8
Rpl10a
Rps15
Rplp2-ps1
Rpl28
Rplp2
TCONS_00002395
Rps21
Rpl24
Rpl29
Nme2
Rps16
Rps14
