# Supplementary material for: Identification and functional analysis of long non-coding RNAs in mouse cleavage stage embryonic development based on single cell transcriptome data
Source: BMC Genomics. 2014 Oct 3;15(1):845. doi: 10.1186/1471-2164-15-845 (PMC4200203; doi:10.1186/1471-2164-15-845)
Supplement: Supplementary file 17 — Additional file 17: Public annotations used in this study. Public annotations used in this study. (DOCX 26 KB) [file 12864_2014_6548_MOESM17_ESM.docx]

| Dataset | Source | Reference | Number of transcripts | Note |
| --- | --- | --- | --- | --- |
| Protein coding transcripts known to RefSeq | The UCSC genome browser; NCBI37/mm9 (Jan, 2013) | [[1](#_ENREF_1)] | 27242 | Extracted only transcripts with an identifier beginning with NM_ |
| Non-coding transcripts known to RefSeq | The UCSC genome browser; NCBI37/mm9 (Jan, 2013) | [[1](#_ENREF_1)] | 13390 | Extracted only transcripts with an identifier beginning with NR_ |
| Non-coding transcripts known to UCSC | The UCSC genome browser NCBI37/mm9 (Jan 2013); The kgTxInfo primary table | [[2](#_ENREF_2), [3](#_ENREF_3)] | 15205 | Extracted transcripts annotated as non-coding |
| Coding transcripts known to UCSC | The UCSC genome browser NCBI37/mm9 (Jan 2013); The kgTxInfo primary table | [[2](#_ENREF_2), [3](#_ENREF_3)] | 43689 | Extracted transcripts annotated as coding |
| Non-coding transcripts known to ENSEMBL | Ensembl NCBI37/mm9 (Jan 2013) | [[4](#_ENREF_4)] | 11432 | Extracted only transcripts with biotype=”lincRNA” or “non-coding” |
| Protein-coding transcripts known to ENSEMBL | Ensembl NCBI37/mm9 (Jan 2013) | [[4](#_ENREF_4)] | 79662 | Extracted only transcripts with biotype=”protein_coding” |
| Non-coding transcripts known to NONCODE3 | From http://www.noncode.org (Jan, 2013; NONCODE3) | [[5](#_ENREF_5)] | 36990 |  |

Public annotations used in this study.

1. Mouse Genome Sequencing C, Waterston RH, Lindblad-Toh K, Birney E, Rogers J, Abril JF, Agarwal P, Agarwala R, Ainscough R, Alexandersson M *et al*: **Initial sequencing and comparative analysis of the mouse genome**. *Nature* 2002, **420**(6915):520-562.

2. Hsu F, Kent WJ, Clawson H, Kuhn RM, Diekhans M, Haussler D: **The UCSC Known Genes**. *Bioinformatics* 2006, **22**(9):1036-1046.

3. Meyer LR, Zweig AS, Hinrichs AS, Karolchik D, Kuhn RM, Wong M, Sloan CA, Rosenbloom KR, Roe G, Rhead B *et al*: **The UCSC Genome Browser database: extensions and updates 2013**. *Nucleic acids research* 2013, **41**(Database issue):D64-69.

4. Flicek P, Ahmed I, Amode MR, Barrell D, Beal K, Brent S, Carvalho-Silva D, Clapham P, Coates G, Fairley S *et al*: **Ensembl 2013**. *Nucleic acids research* 2013, **41**(D1):D48-D55.

5. Bu D, Yu K, Sun S, Xie C, Skogerbo G, Miao R, Xiao H, Liao Q, Luo H, Zhao G *et al*: **NONCODE v3.0: integrative annotation of long noncoding RNAs**. *Nucleic acids research* 2012, **40**(Database issue):D210-215.
